# Patterns in GP appointment systems: a cluster analysis of 3480 English practices

**DOI:** 10.3399/BJGP.2024.0556

**Published:** 2025-07-01

**Authors:** James Scuffell, Stevo Durbaba

**Affiliations:** 1 School of Life Course and Population Sciences, King’s College London, Guy’s campus, London SE1 1UL, UK

**Keywords:** primary health care, remote consultation, telemedicine, appointment and schedules, cluster analysis, health workforce

## Abstract

**Background:**

In response to increasing demand for appointments, UK general practices have adopted a range of appointment systems. These systems vary widely in implementation. These changes have not yet been clearly described.

**Aim:**

To characterise patterns of primary care delivery in English general practices.

**Design and setting:**

Cross-sectional study using NHS Appointments in General Practice data from 3480 English GP practices, totalling 56 million appointments between August and October 2023.

**Method:**

Twelve measures associated with consultation modality, waiting time, clinician type, and triage use were derived. Practices with similar characteristics for those 12 variables were clustered together using an ensemble machine learning approach. Clustering was validated using December 2023 data. The characteristics of each practice grouping were described using 2021 Census and NHS workforce data.

**Results:**

Two main models of care emerged. ‘Routine care’ practices (*n* = 2286) tended towards face-to-face appointments, often delivered by non-GPs with longer wait times. ‘Same-day care’ (*n* = 1194) practices, a third of practices, were more likely to use telephone consultations, deliver care with GPs, and provide same-day appointments. Compared with ‘routine care’ practices, ‘same-day care’ practices were more likely to be in urban areas, had younger populations (mean age 40 years versus 41 years) and employed fewer patient-facing staff in extended roles (all clinical staff except doctors and nurses) (2.0 versus 2.5 full-time equivalents per 10 000 patients registered).

**Conclusion:**

This study identified two dominant models of primary care delivery in England, reflecting differing approaches to managing patient access. These differences could have an impact on continuity of care and equity of access to primary care.

## Introduction

Recently UK primary care has pivoted rapidly towards remote consultations to manage increasing primary care demand. The NHS Long Term Plan required the adoption of ‘digital-first care’ – including online consultations – by 2023/2024. The 2022 Fuller Stocktake Report, written to set a direction for primary care access in the UK, acknowledges that people ‘prioritise different things’, that some need to be seen straightaway whereas ‘others are happy to get an appointment in a week’s time’.^1^ How general practices have responded to these challenges is not clearly understood.

The process of booking GP appointments in England varies significantly between practices. Patients may or may not undergo clinical triage and could be seen by a range of healthcare professionals, including pharmacists, nurses, GPs, and physician associates. Consultations may occur by telephone or face to face, either on the same day or after a waiting period of several weeks. These redesigns of appointment systems although aimed at improving access could have an impact on continuity of care.^2^

An analysis of clinical workload using consultation — rather than appointment — data from 2007 to 2014 demonstrated increasing clinical intensity, longer consultation durations and increased reliance on telephone triage.^3^ Patients in the most deprived areas were found to have more frequent but shorter consultations.^4^ Evaluations of telephone triage occurred before widespread clinician competence in remote consulting,^5^ showing few changes in clinical workload after GP- or nurse-led triage.^6,7^

**How this fits in**

GP practices in the UK are using a wide range of different appointment systems to meet patient demand and improve access. This cluster analysis of NHS appointment data from 56 million appointments and 3480 English practices demonstrates two predominant models of primary care delivery. ‘Same-day care’ practices tend to fulfil appointments on the same day using GP telephone consultations. ‘Routine care’ practices tend to employ non-GP staff members offering face-to-face appointments and longer appointment wait times. ‘Same-day’ care practices had younger and more urban populations.

Previous research characterising appointment systems using routine data have either been conducted at a local geographical scale^8,9^ or in a trial setting,^5^ and all pre-date the widespread use of remote consulting. The differences in how appointment systems are used in various practices — by appointment type, consultation method, and how staff are utilised — have made it harder to classify GP appointment systems using administrative data.

The Appointments in General Practice dataset,^10^ published by NHS England and NHS Digital since 2019, contains detailed information on GP appointments across England and gives new opportunities to describe how primary care is organised. The aim was to use these appointment data to explore differing approaches to delivering primary care in English practices.

## Method

### Description of the Appointments in General Practice dataset

The Appointments in General Practice dataset,^10^ published by NHS England monthly since 2019, contains appointment data for 95.6% of English GP practices, covering 96.4% of registered GP patients.^11^ It is automatically extracted from GP systems. Each appointment is categorised by type (telephone, face to face, home visit, or video); healthcare professional (GP or ‘other practice staff’); appointment status (booked, attended, did not attend); and timing (days between booking and the appointment). Each month, the number of appointments in these categories is recorded at the practice level. GP practices are paid^12^ to accurately record all appointments, using a standard set of standardised appointment types defined by NHS England.^13^

In this study, appointment data from August 2023 to October 2023 was used.^10^ The data was improved by annotating the appointments using the authors' knowledge of primary care and including only practices with high-quality appointment records. Practices were grouped based on similar appointment characteristics using 12 measures of the appointment system.

### Data quality and cleaning

NHS England refers to the Appointments in General Practice data as ‘experimental statistics’^11^ because of differences in data quality across practices. In this study, to ensure that practices included in the sample were representative of English GP practices and had reliable data, the data were cleaned in four stages.

- Practices with <1000 patients or appointment rates >1500 per 1000 per year, were excluded, as they may represent opening or closing practices or those with specialist patient populations.^14^
- Practices were removed where >10% of appointments were not mapped to a standardised category^15^ between August 2023 and October 2023.
- Practices with likely inaccurate appointment coding were excluded.
- Sensitivity analyses were run to compare the characteristics of practices that were included versus those excluded.

Although practices are supported to map appointments to a standard list of appointment categories,^15^ some appointments were inaccurately mapped. To improve quality, one of the authors (a GP) manually reviewed and labelled common appointment combinations as ‘same day’, ‘routine’, ‘triage’, or ‘unknown’. The full methodology is available in Supplementary Information S1 and the annotated combinations are available online.^16^ Practices with <50% of their appointments annotated were excluded, as this was likely because of inaccurate coding.

### Defining measures to differentiate appointment booking patterns

- In this study the authors aimed to group GP practices with similar appointment booking patterns, based on 12 measures based on type of consultation, healthcare professional undertaking the appointment, and how long patients waited for the appointment. These measures are shown in Box 1.

**Box 1.** Definitions of variables used to determine practice clusters

Proportion of appointments	Denominator
With a GP	Total GP appointments
Booked more than one week in advance	Total GP appointments
Telephone appointments	Total GP appointments
Telephone appointments with a GP	Total GP appointments
Same day appointments with a GP	Total GP appointments
Appointments annotated as ‘same-day’	Total annotated appointments
Telephone appointments annotated as ‘same-day’	Total annotated appointments
GP appointments annotated as ‘same-day’	Total annotated appointments
Appointments annotated as ‘routine’	Total annotated appointments
Appointments annotated as ‘routine’ delivered within one day of booking	Appointments annotated as routine
Use of clinical triage	Any recording of clinical triage for a given month, 0 or 1.
Total number of appointments	List size

Data on care home visits and home visits was not included because, in England, these vary more because of sociodemographic factors than appointment-book patterns. A sensitivity analysis was undertaken including care home visits, available in Supplementary Information S2.

For each practice, the average for each measure was calculated from August 2023 to October 2023 and then these measures were scaled to a range between 0 and 1.

### Clustering

Cluster analysis was used to group GP practices with similar appointment booking patterns. To maximise the interpretability of the findings, the authors' chose a priori to split practices into either two or three groups.

Ensemble clustering was applied, an unsupervised machine learning method that creates stable and reproducible clusters by running different clustering techniques on random samples of the data multiple times. The final clustering is determined by a majority vote from the repeated results.^17^

Five clustering methods were used: *k*-means clustering (Lloyd’s method^18^), hierarchical (Ward’s minimum variance method),^19^ hierarchical self-organising maps,^20^ partitioning around medoids,^21^ and Gaussian mixture models using Bayesian Information Criterion. Each method was repeated 100 times, using random samples of 80% of practices, for 400 iterations. Analysis were performed using R 4.2.1 using the diceR package^22^ and the code is available online.^16^

How consistently practices were assigned to a given cluster was measured using the mean adjusted Rand index for each cluster assignment pair.^23^ The authors also tested the clustering method on new data from December 2023, prepared identically to the August to October 2023 dataset. The proportion of practices that remained in the same cluster using the December 2023 data are also reported.

### Descriptive analysis

Using 2022–2023 data the authors compared the characteristics of practices in each cluster using practice registration data,^24^ sociodemographic data from the 2021 Census, and General Practice Workforce data from September 2023.^25^ The study considered practice list size, age of the practice population, attributed index of multiple deprivation (IMD) scores based on patient lower super output area registration in 2019, rural–urban classification, and 2021 Census ethnic group data (White, Black, Asian, Other, Mixed) for the registered population. The study also considered the full-time equivalent (FTE) number of nurses, advanced nurse practitioners, paramedics, and GPs (including locum and training grade).

## Results

### Practice characteristics

**Figure 1.**
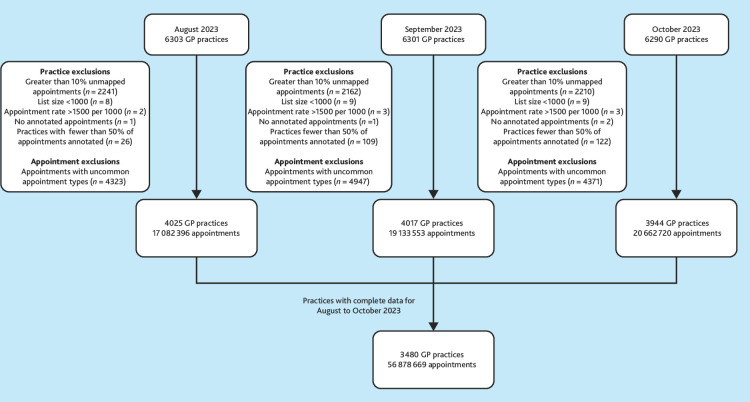
Diagram showing how practices were included in cluster analysis.

Of 6301 English GP practices with appointment data in September 2023, 3480 (55.2%) had sufficient data quality to use for analysis, totalling 56.9 million appointments (Figure 1). The sociodemographic profiles of practices included and excluded from analysis were similar (Supplementary Figure S1, Supplementary Table S1).

Using data from August 2023 to October 2023, practices were grouped into two clusters. The mean adjusted Rand index was 0.25, indicating fair agreement given the number of clusters chosen.^26^ The clustering was also tested using December 2023 data, and 88% of practices were classified in the same cluster (Supplementary Figure S2).

### Description of clusters

Each of the 3480 practices was grouped into two clusters (Table 1).

**Table 1.** Practice characteristics of the 'routine' and ’same-day' clusters, August to October 2023

Practice characteristics (*N*=3480)	Routine cluster (*N*=2286)	Same-day cluster (*N*=1194)
**Proportion of appointments, mean (SD)**		
With a GP	0.48 (0.15)	0.62 (0.13)
Booked more than 1 week in advance	0.35 (0.13)	0.26 (0.11)
Telephone appointments	0.18 (0.14)	0.33 (0.17)
Telephone appointments with a GP	0.11 (0.10)	0.24 (0.14)
Same day appointments with a GP	0.27 (0.12)	0.41 (0.12)
Appointments annotated as ‘same day’	0.16 (0.13)	0.43 (0.19)
Telephone appointments annotated as ‘same day’	0.03 (0.05)	0.16 (0.14)
GP appointments annotated as ‘same day’	0.10 (0.09)	0.32 (0.14)
Appointments annotated as ‘routine’	0.70 (0.19)	0.48 (0.19)
Appointments annotated as ‘routine’ delivered within 1 day of booking	0.35 (0.17)	0.35 (0.17)
Total appointments per 1000 patients	488.49 (128.61)	462.71 (110.36)
**Practices using clinical triage**	1844 (80.7)	900 (75.4)
**Practice list size, mean (SD)**	9784 (6335)	10 377 (7355)
**Region of England**		
East of England	281 (12.3)	79 (6.6)
London	358 (15.7)	273 (22.9)
Midlands	417 (18.2)	276 (23.1)
North East and Yorkshire	387 (16.9)	96 (8.0)
North West	381 (16.7)	217 (18.2)
South East	255 (11.2)	181 (15.2)
South West	207 (9.1)	72 (6.0)
**Proportion of practice population of White ethnic group (2021), mean (SD)**	0.81 (0.21)	0.75 (0.22)
**Index of multiple deprivation quintiles 2019^a^**	2150	1167
1 (most deprived)	403 (18.7)	252 (21.6)
2	420 (19.5)	224 (19.2)
3	416 (19.3)	214 (18.3)
4	444 (20.7)	238 (20.4)
5 (least deprived)	467 (21.7)	239 (20.5)
**Rural–urban status of practice**		
Urban	1894 (82.9)	1054 (88.3)
Rural	392 (17.1)	140 (11.7)
**Age distribution of practice lists, %**		
<20 years	22	23
20–49 years	40	42
50–64 years	20	19
65–79 years	13	12
80 years	5	4

Data are *n* (%) unless otherwise specified.

SD = standard deviation

^a^There are missing data for this characteristic.

Cluster 1 (‘routine care’ cluster) contained 2286 practices with 22.4 million registered patients. These practices were geared toward face-to-face care, delivered by a mixture of GPs and non-GPs, with longer waits for appointments.

Cluster 2 (‘same-day care cluster’) contained 1194 practices with 12.4 million registered patients. These practices, compared with the ‘routine care’ cluster, were more likely to deliver appointments by telephone (33% of appointments versus 18%), appointments with GPs (62% versus 48%), and a higher proportion of same-day appointments (41% versus 27%).

Appointments in the 'same-day care’ cluster were also more frequently annotated as representing same-day care (43% versus 16%) and less likely to be annotated as routine (48% versus 70%). Practices in the ‘same-day care’ cluster had on average slightly larger numbers of registered patients than those in the ‘routine care’ cluster (10 377 versus 9784).

### Sociodemographic characteristics of practices

**Figure 2.**
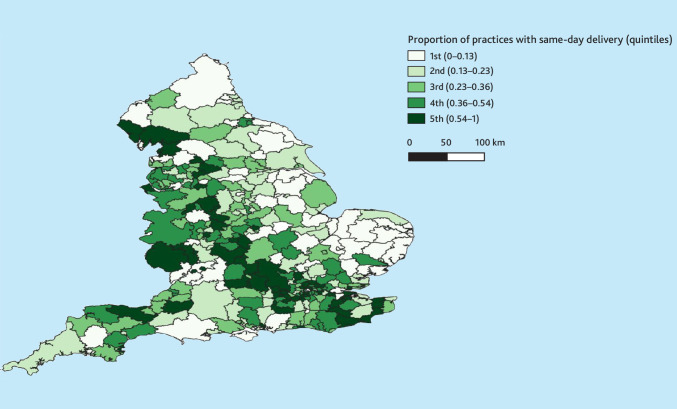
Choropleth map showing the proportions of GP practices within each local authority boundary that are in the same-day cluster. 2022 Local authority boundaries. (https://www.data.gov.uk/dataset/d2724ae1-848d-4b97-baf0-63d769bcf28e/local-authority-districts-december-2022-boundaries-uk-bfe). Office for National Statistics licensed under the Open Government Licence v.3.0. Contains OS data © Crown copyright and database right 2025

‘Same-day care’ practices had slightly younger patient populations than ‘routine care’ practices (mean age 40 years (SD 22.0) versus 41 (SD 22.5); 35% over 50 versus 38%; Box 1). There was strong evidence that ‘same-day care’ practices were more likely to be situated in London (22.9% [273/1194] versus 15.7% [358/2286] of routine care practices) or the Midlands (23.1% (276/1194) versus 18.2% [417/2286]), and less likely to be in rural areas (11.7% [140/1194] versus 17.1% [392/2286], Figure 2). Both ‘routine care’ and ‘same-day care’ practices were equally likely to serve deprived populations based on 2019 IMD estimates.

### Workforce characteristics

There were notable differences in workforce characteristics between ‘routine care’ and 'same-day care’ practices (Table 2). On average, ‘routine care’ practices had more staff, a mean of 14.1 FTE staff per 10 000 patients compared with 13.2 FTEs per 10 000 for ‘same-day care’ practices. ‘Routine care’ practices employed more administrative and clinical staff in extended roles (11.6 versus 11.1 FTEs per 10 000 for admin staff, and 2.5 versus 2.0 FTEs per 10 000 for clinical staff in extended roles). ‘Routine care’ practices also employed more practice nurses (1.8 versus 1.6 FTEs) and advanced nurse practitioners (0.6 versus 0.4 FTEs).

**Table 2.** Workforce characteristics of routine and same-day cluster practices^a^

Full-time equivalent staff per 10 000 patients, mean (SD)*N* = 3317)		
Practice characteristics (N = 3317*)*	Routine cluster (*N* = 2182)	Same-day cluster (*N* = 1135)
**GP**	4.4 (1.9)	4.5 (1.8)
**All nurses**	2.7 (1.7)	2.2 (1.4)
Practice nurses	1.8 (1.1)	1.6 (0.9)
Advanced nurse practitioners	0.6 (0.9)	0.4 (0.7)
**Clinical staff in extended roles^b^**	2.5 (2.5)	2.0 (2.3)
**Pharmacists**	0.2 (0.5)	0.2 (0.5)
**Paramedics**	0.1 (0.4)	0.1 (0.2)
**Physician associates**	0.1 (0.3)	0.1 (0.4)
**Non-patient facing care**	11.6 (4.1)	11.1 (4.1)

^a^Data from General Practice Workforce data as at 30 September 2023, https://digital.nhs.uk/data-and-information/publications/statistical/general-and-personal-medical-services. Data were available for 3317 of 3480 practices (95.3%).

^b^Clinical staff in extended roles are all patient-facing staff excluding doctors and nurses.

SD = standard deviation.

In contrast, ‘same-day care’ practices were marginally more likely to employ GPs (4.5 versus 4.4 FTEs per 10 000 patients). There were no substantial differences in the employment of pharmacists, paramedics, or physician associates between the two groups of practices.

## Discussion

### Summary

Since the COVID-19 pandemic a wider range of primary care delivery methods have proliferated in the UK. This study, to the best of the authors' knowledge, is the first to identify patterns of practising in England and to compare the characteristics of practices with differing working models.

Despite substantial heterogeneity this study identified two main clusters: some prioritising same-day appointments by telephone with a GP; some using nurses and advanced nurse practitioners to deliver face-to-face care, with longer wait times for appointments. The practice populations served by ‘same-day care’ practices tended to serve younger, urban, more ethnically diverse areas — although deprivation levels were similar in both groups. Practices in both clusters were equally likely to record use of clinical triage. Overall, ‘same-day care’ practices employed fewer clinical and admin staff compared with those delivering a more traditional general practice model of care.

### Strengths and limitations

The data were cleaned and clustering variables selected based on the authors' knowledge of English primary care. A range of clustering techniques were used to identify two predominant working models, including over 56 million consultations for 3 months of 2023 to ensure month-to-month reliability of appointment patterns. The clusters were validated with December 2023 data. The misclassification error of 12% could reflect changes in the appointment book over several months, or it may demonstrate a spectrum of ways of working in general practice in England that causes overlapping in the clusters. It may also indicate the variables chosen for clustering did not fully capture the ways of working of GP practices. Improving the quality of data – which should happen with increased use of the dataset — may improve the reliability of the clustering.

This study limited the number of clusters to balance precision with interpretability.^27^ With increased sharing of telephony and online data — required in NHS England’s 2024–2025 contract^28^ — it may be possible to assess the impact of appointment book changes on patient care.

The study used contemporaneous GP workforce data to compare ‘same-day care’ and ‘routine care’ practices. Although differences were seen, it is difficult to establish causality from these ecological panel data, where patient and clinician factors may also be at play. Although ‘same-day care’ practices had a slightly younger patient population, age-stratified appointment rates were not available and it was not possible to compare clinical workload between practice clusters. The two groups of practices may cater for patients with different expectations of care or clinical complexity. As these data do not describe the reason for appointments, it was not possible to compare the acute and routine workload between practices. Reverse causality may also be at play, as appointment books may be designed to match the available primary care workforce. A prospective study would help to disentangle these challenges.

Although the sample of practices was large and representative of English GP practices, over a third of practices were excluded because of data quality. Engaging with these practices could improve data usage, allowing GPs and commissioners to use these data to make informed organisational decisions.

### Comparison with existing literature

Previous studies have examined appointment patterning at a patient level^3,29^ or explored how primary care delivery methods could theoretically have an impact on workload.^30^ The dataset used in the current study has been used before to describe how crude appointment rates are associated with workforce and population characteristics. That study also described evidence of a substitution effect between GPs and nurses.^14^ The current study extends these findings, suggesting that there may be substitution by clinician, consultation modality, and also the lead time for an appointment.

### Implications for research and practice

Increasing pressures for general practice appointments have coincided with an increase in telephone consultations since the COVID-19 pandemic. This study identifies that GP practices that tend to use telephone consultations also deliver care on the same day and with a GP. About a third of GP practices in England follow this ‘same-day care’ approach.

National conversations about GP access – which tend to prioritise same-day care to increase patient satisfaction with GP services – may be more complicated. Practices use different consultation modalities and other members of the primary care workforce to maximise access. Increasing the supply of appointments without regard to consultation modality or healthcare professional may not improve health or patient-recorded outcomes.^31^ It may also have an impact on continuity of care, and not be sensitive to the changing needs of people living with multimorbidity. A prospective study of these data is now required to consider whether there is a causal relationship between same-day appointment delivery, patient outcomes, and continuity of care.^32^

It is notable that ‘same-day care’ practices also tend to employ fewer FTE admin and clinical staff, despite similar appointment rates. ‘Same- day care’ practices were slightly larger on average, possibly benefiting from administrative economies of scale. Further patient-level research should consider whether patients face different levels of access in these two models of care, and whether there are differences in clinical workload.

This study identified two predominant models of care delivery in English general practices, each with different underlying practice populations and approaches to staff employment. Improving data accuracy and conducting patient-level studies will help GPs better understand the implications for clinical workload, patient outcomes, and continuity of care.

## Data

NHS Appointments in General Practice data is available at https://digital.nhs.uk/data-and-information/publications/statistical/appointments-in-general-practice. The statistical analytical code is available on Figshare https://figshare.com/articles/online_resource/Appointment_book_patterns_in_English_primary_care_cluster_analysis_of_3480_practices_-_supplementary_data/26780233 (doi:10.6084/m9.figshare.26780233.v1)
